# Validity and Reliability of the Tunisian Arabic Functioning Assessment Short Test (FAST) and Functional Outcome Factors in a Sample of Patients with Bipolar Disorder

**DOI:** 10.2174/0117450179361739250203043410

**Published:** 2025-02-07

**Authors:** Hend Jemli, Uta Ouali, Ons Maatouk, Ahlem Hajri, Azza Ben Cheikh Ahmed, Amina Aissa, Rabaa Jomli

**Affiliations:** 1 Department Psychiatry A, Razi Hospital, Manouba, 2010, Tunisia; 2 Faculty of Medicine of Tunis, University of Tunis El Manar, Tunis, 1068, Tunisia; 3 Outpatient Psychiatry Department, Razi Hospital, Manouba, 2010, Tunisia

**Keywords:** Validation, Questionnaire, Bipolar disorders, Quality of life, Disability, FAST, Functioning

## Abstract

**Background:**

Bipolar disorders negatively impact functional outcomes and, consequently, prognosis. The Functioning Assessment Short Test is a reliable tool to evaluate functional outcomes in people with bipolar disorders.

**Aim:**

The aim of the study was to conduct a cross-cultural validation of the Functioning Assessment Short Test (FAST) and to explore correlations between functional impairment and sociodemographic and clinical variables.

**Methods:**

A cross-sectional study was carried out in a population of 60 bipolar patients and 60 healthy controls. The scales administered were the Global Functioning Assessment (GAF), the World Health Organization Quality Of Life-Bref (WHOQOL-BREF), the FAST, and a questionnaire containing sociodemographic and clinical variables. The validation study was based on face and content validity, reliability, and construct validity.

**Results:**

The face and content validity were satisfactory. The internal consistency obtained was high, with a Cronbach’s alpha of 0.785. All six FAST domains had significant correlations with each other and with the total score. The FAST assessment at baseline and week 2 were highly correlated (*p*>0.05), and the intraclass correlation coefficient was 0.998, indicating high test-retest reliability. The FAST total score was negatively and significantly associated with GAF (rho=-0.788, *p*<0.001) and WHOQOL-BREF scores, suggesting good concurrent validity. The total FAST scores were significantly lower in controls as compared with bipolar patients (*p*<0.001), with a cut-off at 26. Functional impairment was significantly associated with the following variables: low educational level, living alone, early age at onset, number of depressive episodes, and treatment associations (mood stabilizers and antipsychotics).

**Conclusion:**

The Tunisian Arabic version of the FAST demonstrated satisfactory psychometric properties and could be used to assess specific domains of functional impairment in people living with bipolar disorders and may be instrumental in implementing psychosocial and rehabilitation interventions.

## 1 INTRODUCTION

The World Health Organization classifies Bipolar Disorder (BD) as one of the ten most disabling illnesses [1]. Their chronic course, early onset, and high risk of suicide could explain the high rates of morbidity and mortality [2, 3]. Bipolar disorders are associated with functional variability ranging from complete remission to a state of disability [4]. The concept of functional impairment involves several domains and areas of life, such as autonomy, academic or professional abilities, cognitive functioning, financial management, social integration, interpersonal relationships, and leisure activities.

Although a multitude of therapeutic strategies to reduce the frequency and duration of manic and depressive episodes exist, patients often do not achieve full remission with the persistence of sub-threshold mood symptoms. In addition, clinical remission seems to be dissociated from functional recovery in patients with BD. In contrast with earlier studies, only one-third of bipolar patients regain their functioning level prior to inpatient admission for relapse [5].

The negative impact of bipolar disorder on socio-occupational functioning is well established [6]. It can affect professional status and work attendance, ranging from absenteeism to a prolonged inability to find and keep a job [7]. Regarding social life, a significant proportion of patients with BD experience interpersonal relationship difficulties, face discrimination and stigma, and lack social support [8]. In terms of clinical correlates, higher disabilities in patients with BD are associated with cognitive impairment during the inter-episodic period. Specific impairments in executive function and verbal memory have been noted in bipolar disorder.

In clinical research, it is essential to dispose of standardized and reliable disability assessment tools [9]. Several functioning assessment scales have been developed for this purpose, such as the Global Functioning Scale (GFS) [8], the Work and Social Adjustment Scale (WSAS) [10], and the Life Functioning Questionnaire (LFQ) [11]. However, these instruments are not specific to mood disorders and, therefore, fail to accurately characterize disability associated with BD.

Consequently, the Functioning Assessment Short Test (FAST) was designed in 2007 by Rosa *et al*. as part of the Bipolar Disorder Program in Barcelona for the clinical evaluation of functional impairment of patients suffering from bipolar disorders [12]. It is a brief, ergonomic interview-administered questionnaire that explores 6 key areas of functioning: autonomy, occupational functioning, cognitive functioning, financial issues, interpersonal relationships, and leisure time.

Furthermore, although researchers are increasingly interested in determinants of impaired functioning in people with BD, few studies were made on the subject in the MENA region. One study was carried out in Egypt and evaluated quality of life among forty patients with BD and found a significant correlation between adequacy of monthly income and total quality of life [13]. One literature review on bipolar disorder characteristics in various Arab countries, such as Lebanon, Qatar, Tunisia, and Oman, included 25 publications in total [14]. The study focused on epidemiology, clinical characteristics, and service utilization with little to no mention of functional outcome [14].

The aim of this study was to evaluate the psychometric properties of the Tunisian version of the “Functioning Assessment Short Test” in a sample of individuals with bipolar disorders and to determine factors associated with functional outcomes in this population.

## 2 METHODS

### 2.1 Design and Participants

This study has a cross-sectional design and was conducted between February 2022 and June 2022.

Sixty outpatients were consecutively recruited at Razi Psychiatric Hospital in Tunis/ Tunisia. Patients who fulfilled the following criteria were included: (i) clinical diagnosis of Bipolar Disorder type I or type II according to DSM 5, (ii) 18 years or older, (iii) euthymic at the time of the study with scores < 8 on the Hamilton Depression Rating Scale and < 6 on the Young Mania Rating scale), and (iv) no history of a manic or depressive episode nor psychiatric inpatient admission within the three months prior to study intake. Patients with comorbid intellectual deficiency, major cognitive impairment, or physical disability that could significantly impact their functioning were not included. The sample size was determined based on previous validation studies of the FAST.

Sixty controls were recruited among the visitors of Kassab Orthopedics Hospital in Tunis/Tunisia and were matched for sex and age. Individuals who fulfilled the following criteria were included: (i) no current depressive or (hypo)manic episode according to a screening with the Structured Clinical Interview for Diagnosis and Statistical Manual of Mental Disorders-Fourth Edition (SCID), (ii) no personal or first-degree relatives’ history of psychiatric disorders, (iii) no personal history of intellectual deficiency or major cognitive impairment, and (iv) no physical disability that could significantly impact their functioning.

### 2.2 Assessments and Procedures

Information was gathered on socio-demographic and clinical variables, including age, sex, marital status, education, employment status, bipolar disorder type and polarity, age of onset, number of depressive and manic episodes, treatment resistance, prior psychotic features, number and duration of hospitalizations, number of suicide attempts, current treatment, treatment compliance and comorbid psychiatric disorders, such as substance use and anxiety disorders.

The Global Assessment of Functioning Scale (GAF) and the brief version of the World Health Organization Quality of Life scale (WHOQOL-BREF) were chosen to assess concurrent validity. The first researcher recorded socio-demographic and clinical variables and administered the FAST. A second researcher administered the GAF and WHOQOL-BREF. The two interviewers were blinded to each other. Test-retest reliability was checked two weeks after the original interview in 25 patients.

### 2.3 The Functioning Assessment Short Test (FAST)

The FAST is an interview-administered instrument designed to be used by a trained clinician [12]. The studied time frame refers to the last two weeks before the assessment. It comprises 24 items divided into 6 specific areas of functioning: autonomy, occupational functioning, cognitive functioning, financial issues, interpersonal relationships, and leisure activities. All items are rated on a four-point Likert scale. The global score is obtained by adding the scores of each item. The higher the score, the more severe the functional impairment. The original Spanish version of the FAST has been translated into English, Italian, Portuguese, Chinese, Finnish, and Turkish.

### 2.4 World Health Organization Quality of Life-brief Version (WHOQOL-BREF)

The ‘WHOQOL-BREF’ was developed by the World Health Organization in 1998 to provide a succinct and ergonomic scale for assessing quality of life [15]. It is derived from the World Health Organization Quality of Life-100 questionnaire (WHOQOL-100), which was considered too long for clinical use [16].

The WHOQOL-BREF consists of 26 items. It includes two global items and 24 items divided into four general domains: physical health, psychological health, social relationships and quality of the environment [15]. Item 1 and item 2 assess global appreciation of quality of life and global health satisfaction, respectively.

The WHOQOL-BREF has four types of response scales rated from 1 to 5, allowing assessment of intensity (Not at all-Extremely), ability (Not at all-Completely), frequency (Never-Always), or assessment of satisfaction (Very dissatisfied/Very poor-Very satisfied/Very good) [17].

The WHOQOL-BREF has been translated and validated in more than ten languages, including Arabic [18]. We used the Tunisian version as the external reference questionnaire for the study of the convergent validity of the FAST.

### 2.5 Global Assessment of Functioning (GAF)

Global Assessment of Functioning (GAF) is a single measure of overall psychosocial impairment caused by mental factors ranging from 0 to 100. It constitutes Axis V of the Diagnostic and Statistical Manual of Mental Disorders, Fourth Edition, Revised Text DSM-IV-TR. The clinician chooses to give the patient a score from 0 to 100, which corresponds to his or her overall functioning (psychological, professional, and social). The higher the score, the more satisfactory the patient's functioning [8].

### 2.6 Validation Procedure

After obtaining authorization from the original authors, the English version was translated into Tunisian Arabic and back-translated to English according to Brislin’s back-translation method.

The preliminary version was evaluated by a committee of experts to analyze the clarity, relevance, and discrimination of each item. The revised version was later presented to a pre-test population of twenty patients with bipolar disorders. Changes were made based on the recommendations of the experts and participants.

### 2.7 Statistical Analysis

Statistical analysis was performed using SPSS for Windows- Version 26.0. Internal consistency was measured using Cronbach’s alpha and the item-scale correlation using the Spearman correlation coefficient. Test-retest reliability was assessed using the intra-class correlation coefficient and the Wilcoxon rank test using two matched samples.

Concurrent validity was measured by Spearman’s correlation coefficient to examine correlations between GAF and FAST (total score and dimension scores) and WHOQOL-BREF and FAST (total score).

Discriminant validity was measured by the Mann-Whitney coefficient to compare the FAST scores between patients and control subjects. The cut-off value between patient and control groups was determined by the Receiver Operating Characteristic (ROC) curve analysis.

For the univariate analysis, we examined the correlations of socio-demographic and clinical variables with impaired functioning in the patient group according to the cut-off score of the FAST (dependent variable). The following statistical tests were used: Fisher's exact test, Chi-squared test, and Mann-Whitney test. The significance level was set at 0.05.

Multivariate binary logistic regression was used to identify independent factors that might influence patient functioning according to the FAST score (dependent variable). We included variables with an acceptable significance level in a multivariate logistic regression model as well as variables identified in the literature as predictors of impaired functioning in people with BD [19, 20]. The degree of dependence between impaired functioning and the various variables was expressed by the Odds Ratio (OR). The confidence interval of the Odds Ratio was set at 95%.

## 3 RESULTS

### 3.1 Sociodemographic and Clinical Characteristics

Sixty patients with BD (BP I: fifty-three, BP II: seven) participated in the study. Males comprised 58% of the study population, with a sex ratio of 1.4. The mean age of participants was 45.43 ± 12.03 (ranging from 23 to 70 years). Table **1** shows the sociodemographic and clinical features of both patient and control groups.

### 3.2 Reliability Analysis

The internal consistency obtained was high, with a Cronbach’s alpha of 0.785. The Cronbach’s Alpha in each functioning domain also indicated good internal consistency, ranging from 0.815 to 0.735, except for leisure time (0.590) (Table **2**).

All six FAST domains had significant correlations with each other and with the total score (*p*<0.001). The statistical correlation was strongest between the first two functioning domains: autonomy and professional activity (Table **3**).

The intraclass correlation coefficient was 0.998, indicating high test-retest reliability (Table **2**).

Following the comparison of the patients’ mean scores between baseline and week 2 using the Wilcoxon test, we did not find any statistically significant difference in the mean values between T0 and T1 for the total FAST score as well as for the scores of the different FAST domains (*p*>0.05).

### 3.3 Construct Validity Analysis

Concurrent validity based on functional impairment according to the GAF scale showed a highly significant, negative, and linear correlation with the FAST scale (rho= -0,788, *p*<0.001). These results indicate that patients with impaired functioning assessed using the FAST obtained lower scores on the GAF scale. The GAF score also had negative correlations to each specific FAST dimension (Table **4**).

The correlation between the total FAST score and domains of the WHOQOL-BREF was significant and negative: FAST and item 1 on quality of life (rho=-0.533, *p*<0.001), FAST and item 2 on general health (rho=-0.521, *p*<0.001), FAST and physical health dimension (rho=-0.635, *p*<0.001), FAST and psychological health dimension (rho=-0.663, *p*<0.001), FAST and social relationships dimension (rho=-0.582, *p*<0.001), and FAST and environmental health dimension (rho=-0.489, *p*<0.001).

To evaluate the discriminative effect of the FAST, the total scores of patient and control groups were compared. The total score of patients with BD (35.98) was significantly higher than controls (16.5) (*p*<0.001). The mean FAST dimensions scores of patients were approximately twice the controls’ scores (Table **5**).

The optimal cut-off value was calculated using the Receiving Operating Characteristic (ROC) curve. The cut-off value of the FAST was 26 (sensitivity of 73% and specificity of 92%) to differentiate between patients and control subjects. The area under the ROC curve (AUC) was 0.886 (*p*<0.001).

To construct the multivariate logistic regression model, we included the six variables correlated with impaired functioning according to the univariate analysis, as well as the following variables identified in the literature: total number of episodes, number of depressive episodes, and number of hospitalizations. The results of the multivariate regression are detailed in Table **6**. A lower educational level, living alone, age of onset, number of depressive episodes, and treatment association of a mood stabilizer and antipsychotics were all independently correlated with functional impairment.

## 4 DISCUSSION

Our results found that the Tunisian Arabic version of the FAST had satisfactory psychometric properties in Tunisian adults with bipolar disorders. The Tunisian Arabic dialect was chosen instead of Standard Arabic, given the strong relationship between functioning appreciation and the socio-cultural context.

Our finding showed the FAST had a high internal consistency, with a Cronbach’s alpha of 0.794, which is similar to other validated versions [21, 22].

Our study showed positive and significant correlations between the six domains of the Tunisian version of the FAST. To our knowledge, item-total correlations between FAST dimensions were not assessed in previously developed cross-cultural validations of the FAST.

All six functioning FAST dimensions had significant positive correlations with the total score, ranging from 0.427 to 0.855. The correlations between the total FAST score and the dimensions of leisure activities and financial issues were statistically weaker. This could be explained by the Tunisian socio-cultural context, where leisure activities do not hold a prominent place in everyday life, as well as the perception of functioning. Moreover, finances are usually managed by the head of the family with little to no contribution from other family members.

The intra-class correlation coefficient for all items was 0.998 (*p*<0.001). The value of the intra-class correlation coefficient for the different dimensions of the FAST varied between 0.985 and 0.993, indicating high test-retest reliability, which is in line with earlier findings [23, 24].

The GAF is a widely used instrument to evaluate functioning. In our study, the FAST total score was significantly and negatively associated with the GAF, which was predictable. Understandably, a high GAF score indicates good functioning, whereas a high FAST score reflects a great disability.

To increase the quality of our validation work, we used a second external validator, the WHOQOL-BREF, in its Tunisian version, which evaluates the quality of life. Quality of life is a complex concept encompassing functioning, state of health, and well-being [25, 26].

In our study, we found a significant negative but moderate correlation between the total FAST score and all the WHOQOL-BREF components. Functioning and quality of life are two overlapping yet different concepts which may limit the correlation between the two scales. Additionally, the FAST is an interview-administered scale, whereas the WHOQOL-BREF is a self-rated scale. Discrepancies between subjective *versus* objective ratings of functioning have been previously reported in research studies [23].

With regards to discriminant validity, patients with BD had more severe functional impairment compared to controls, which is consistent with previous findings [21, 24-27]. The cutoff value of 26 could discriminate between patients and controls, which is considered higher than the original Spanish version. The mean FAST score in the control group was 16.5, and the mean score in the patient group was 34.67. The cutoff value is, therefore, necessarily higher than 17. However, such findings are probably not a useful feature for clinical use because the FAST is not a screening tool for BD.

Multiple regression analysis found a significant association between the highest level of education and functioning (AOR=1.74 [95% CI 1.14 - 3.65]). In a Tunisian research study on a sample that included patients with BD type 1 in clinical remission, a primary school level was a predictor of a higher FAST score [27].

Living alone was associated with functional decline in our study (AOR=1.53 [95% CI 1.36 - 3.60]). Similarly, in a study published in 2022 investigating the relationship between social support, resilience, and psychosocial functioning, social isolation was significantly correlated with all domains of the FAST, particularly with the relationship domain [28]. This finding seems applicable to the Tunisian socio-cultural context, according to a study on the role of family caregivers of Tunisian patients with BD in treatment management and the promotion of well-being [29].

The early age of onset is a risk factor for functional decline, which is consistent with previous research data [30]. Given the chronic course of bipolar disorders, diagnosis of bipolar disorder at a younger age would imply a longer duration of the illness [31], an increase in the total number of episodes [32], and neurodevelopmental effects [33], and therefore impaired cognitive functioning. However, there is no consensus on the method of identifying the age of onset of BD [34].

Multiple drug associations, particularly the association of a mood stabilizer and an antipsychotic drug for the treatment of bipolar disorder, was associated with significantly more functional impairment in our study (AOR=2.23 [1.02 - 2.14]). The association of various drugs increases the risk of side effects [35], poor therapeutic compliance [36], and care expenses [37]. For all the above reasons, the use of several treatment drugs could be perceived as a burden for patients with BD and hinder their socio-occupational inclusion [38].

The number of depressive episodes was correlated with a higher FAST score (AOR=3.49 [1.77 - 4.65]). Depression symptoms have been extensively studied as a predictor of impaired functioning in patients treated for bipolar disorder [39]. The relationship between the number of depressive episodes and functional decline is thought to exist both for global functioning and for its different domains [25]. In a meta-analysis using the FAST to assess the functioning of euthymic patients with BD, 13 studies found that subclinical depression symptoms were the major factor associated with impaired functioning [4]. Some studies have found that residual depressive symptoms were correlated with cognitive decline, particularly in executive functions [40]. The relationship between functioning and depression symptoms is thought to be bidirectional; depression alters functioning, and functional decline is a risk factor for depression [41].

### 4.1 Strengths and Limitations

Our study is one of the very few assessing functional outcomes in patients with bipolar disorder in the MENA region, highlighting the importance of acknowledging cultural specificities in understanding and evaluating functioning.

Data from the patient group were collected by two different clinicians. The first evaluator administered the FAST, and the second administered both the GAF and the WHOQOL-BREF, thus guaranteeing the independence of the measures and further supporting the validity of the Tunisian Arabic FAST.

The inclusion of a control group in addition to BD patients enabled the assessment of the FAST discriminant validity.

The main limitation of our study is the inclusion of patients consulting at a tertiary care center with a history of one or multiple hospitalizations, and, hence, more severely affected by BD. Therefore, the sample is not representative of the whole spectrum of bipolar disorders with various clinical severities. Moreover, internal validity was not investigated due to the relatively small sample size.

We did not include an assessment of psychotherapy interventions in the survey and their effect on functioning, given the lack of access to psychotherapy in Tunisia.

## CONCLUSION

The Tunisian version of the Functioning Assessment Short Test has shown good validity and reliability in individuals with BD and could be used in clinical and research settings. Our study has shown that impaired functioning was particularly frequent in individuals with persistent sub-syndromal depression and multiple drug associations. Therefore, it is important for clinicians to specifically identify these factors to improve functional outcomes and quality of life.

## Figures and Tables

**Table 1:** Sociodemographic features of the study groups.

-	**Patients** **N=60** **n (%)**	**Controls** **N=60** **N (%)**	**Patients** **N=60** **Mean Score**	**Controls** **N=60** **Mean Score**
**Age**	-	-	45.43±12.03	45.77±13.82
**Gender**	-		-	-
Male	35 (58%)	35 (58%)	-	-
Female	25 (42%)	25 (42%)	-	-
**Relationship status**	-		-	-
Single	27(47%)	10 (17%)	-	-
In a relationship	21 (37%)	45 (75%)	-	-
Divorced	8 (14%)	3 (6%)	-	-
Widowed	1 (2%)	1 (2%)	-	-
**Highest completed level of education**	-		-	-
Primary school	23 (38%)	6 (10%)	-	-
High school	24 (40%)	24 (40%)	-	-
University	13 (22%)	30 (50%)	-	-
**Employment status**	-		-	-
Regular work activity	8 (13%)	29 (48%)	-	-
Irregular work activity	12 (20%)	6 (10%)	-	-
Sick leave	13 (22%)	3 (5%)	-	-
Unemployed	22 (37%)	11(18%)	-	-
Other	5 (8%)	11 (18%)	-	-
**Living situation**	-		-	-
Alone	5 (8%)	1 (2%)	-	-
With close family members	52 (87%)	45 (90%)	-	-
With enlarged family members or friends	3 (5%)	5 (8%)	-	-
**Socio-economic level**	-		-	-
Low	22 (37%)	15 (25%)	-	-
Middle	33 (55%)	38 (64%)	-	-
High	5 (8%)	7 (11%)	-	-
**Bipolar disorder type**	-	-	-	-
BD I	53 (88%)	-	-	-
BD II	7 (12%)	-	-	-
Age of onset	-	-	26.98±8.44	-
Number of total episodes	-	-	4.00±3.98	-
Depressive episodes	-	-	2.23±1.86	-
Manic episodes	-	-	3.54±3.54	-
Number of hospitalizations	-	-	4.25±2.12	-
**Medication**	-	-	-	-
Mood stabilizor	56 (93%)	-	-	-
First generation antipsychotic	11 (18%)	-	-	-
Second generation antipsychotic	38 (63%)	-	-	-
Benzodiazepines	35 (58%)	-	-	-
Hypnotics	7 (12%)	-	-	-
Antidepressants	3 (5%)	-	-	-

**Table 2:** FAST: mean score and validity tests with breakdown into its five dimensions.

-	**Mean Score**	**Cronbach’s Alpha**	**Item-total Correlation Score**	**Intra-class Correlation Score**
**Autonomy**	4.55	0.815	0.835*	0.993**
**Occupational functioning**	8.6	0.873	0.855*	0.997**
**Cognitive functioning**	6.81	0.820	0.687*	0.993**
**Financial issues**	3.46	0.811	0.427*	0.997**
**Interpersonal relationships**	8.05	0.735	0.810*	0.998**
**Leisure activities**	4.5	0.590	0.480*	0.985**
**Total score**	36	0.794	-	0.998**

Note: *Correlation between total score and dimension, *p*<0.001

**p<0.001

**Table 3:** item-total correlation between FAST dimensions.

-	**Autonomy**	**Occupational Functioning**	**Cognitive Functioning**	**Financial issues**	**Interpersonal Relationships**	**Leisure Activities**
**Autonomy**	1.000	-	-	-	-	-
**Occupational functioning**	0.676 (*p*<0.001)	1.000	-	-	-	-
**Cognitive functioning**	0.574 (*p*<0.001)	0.457 (*p*<0.001)	1.000	-	-	-
**Financial issues**	0.190 (p=0.145)	0.214 (p=0.099)	0.222 (p=0.088)	1.000	-	-
**Interpersonal relationships**	0.598 (*p*<0.001)	0.642 (*p*<0.001)	0.405 (p=0.001)	0.297 (p=0.021)	1.000	-
**Leisure activities**	0.455 (*p*<0.001)	0.289 (p=0.025)	0.349 (p=0.006)	0.155 (p=0.235)	0.233 (p=0.073)	1.00

**Table 4:** Correlations between GAF score and FAST dimensions score.

-	**Rho**	**p**
**Autonomy**	-0.702	<0.001
**Occupational functioning**	-0.689	<0.001
**Cognitive functioning**	-0.518	<0.001
**Financial issues**	-0.375	0.003
**Interpersonal relationships**	-0.585	<0.001
**Leisure activities**	-0.388	0.002

**Table 5:** Comparison of FAST dimensions scores between patients and control subjects.

-	**Control Subjects**	**Patients**	**P**
**Autonomy**	2.4	4.55	<0.001
**Occupational functioning**	2.43	8.6	<0.001
**Cognitive functioning**	3.5	6.82	<0.001
**Financial issues**	1.7	3.47	<0.001
**Interpersonal relationships**	3.95	8.05	<0.001
**Leisure activities**	2.52	4.5	<0.001

**Table 6:** Results of the multivariate regression model of FAST.

-	**Adjusted Odds Ratio (AOR)**	**p**	**Confidence Interval (CI) 95%**
**Age**	1,2	0,32	[0,70 – 2,38]
**Highest completed level of education**	1,74	0,03	[1, 14 – 3,65]
**Living situation (living alone)**	1,53	0,01	[1, 36 – 3,60]
**Socio-economic level**	1,12	0,26	[0,12 – 3,95]
**Age of onset**	1,63	0,02	[1,55 – 2,83]
**Mood stabilizer and antipsychotic treatment association**	2,23	0,01	[1,02 – 2,14]
**Total number of episodes**	0,8	0,30	[0,93 - 1,77]
**Number of depressive episodes**	3,49	0,04	[1,77 - 4,65]
**Number of hospitalizations**	1,32	0,23	[0,53 – 1,88]

## Data Availability

The data sets used and/or analysed during this study are available from the corresponding author [H.J] upon request.

## LIST OF ABBREVIATIONS

BD: = Bipolar Disorder
GFS: = Global Functioning Scale
WSAS: = Work and Social Adjustment Scale
LFQ: = Life Functioning Questionnaire
ROC: = Receiver Operating Characteristic
GFS: = Global Functioning Scale
FAST: = Functioning Assessment Short Test
GAF: = Global Assessment of Functioning

## STANDARDS OF REPORTING

STROBE guidelines were followed.
